# Stem cell-based strategies for intervertebral disc regeneration in degenerative microenvironments: challenges and solutions

**DOI:** 10.3389/fcell.2025.1719332

**Published:** 2025-12-10

**Authors:** Honglin Wang, Aoshuang Xu, Wei Hua, Junling Mao, Jiyao Zhang, Xiaobo Ma, Lin Lu

**Affiliations:** 1 Department of Orthopedics, Renmin Hospital of Wuhan University, Wuhan, China; 2 Institute of Hematology, Union Hospital, Tongji Medical College, Huazhong University of Science and Technology, Wuhan, China

**Keywords:** intervertebral disc, low back pain, MSC, regenerative medicine, microenvironment, niche, extracellular vesicles (EVs), disc progenitor cells

## Abstract

Low back pain (LBP) is a common musculoskeletal disorder, and its pathological basis is closely related to intervertebral disc degeneration (IVDD). Although commonly used conservative treatments and surgical interventions can alleviate symptoms, they are difficult to fundamentally delay or reverse the process of IVDD. In recent years, biological treatment strategies centered on cell therapy, targeting the initiating mechanisms of IVDD, have provided new directions for the fundamental treatment of this disease. Among them, mesenchymal stem cells (MSCs) are regarded as ideal candidate cell types for achieving intervertebral disc regeneration due to their immunomodulatory properties and multidifferentiation potential. The discovery of endogenous stem cells within the intervertebral disc further reveals the tissue’s own repair potential. As the “soil” upon which “seed” cells depend for survival, the intervertebral disc microenvironment, composed of cell niches and their surrounding biochemical and physical factors, plays a key regulatory role in the proliferation, differentiation, and functional expression of stem cells and endogenous cells. However, the intervertebral disc is naturally in extreme conditions such as low oxygen, low nutrition, acidic pH, and high mechanical load. This microenvironment further deteriorates during the degeneration process, not only severely affecting the survival and function of resident cells but also posing severe challenges for stem cell transplantation therapy. This article systematically reviews the characteristics of the intervertebral disc microenvironment under physiological and degenerative states, focusing on the impact of the degenerative microenvironment on the biological behavior of stem cells. It summarizes key strategies for enhancing MSC adaptability and therapeutic efficacy and proposes standardized parameters for optimizing clinical applications, aiming to provide a theoretical basis and path support for advancing the clinical translation of stem cell therapy in intervertebral disc regeneration.

## 1 Introduction

Low back pain (LBP) is a common musculoskeletal disorder, affecting over 500 million people globally, with a trend towards younger onset age. It has become a significant public health issue that imposes a heavy socio-economic burden (Ma et al., 2025). Its pathological basis is primarily associated with intervertebral disc degeneration (IVDD) (Liu et al., 2025). The hallmarks of IVDD include a reduction in nucleus pulposus cells, degradation of the extracellular matrix (ECM), activation of an inflammatory microenvironment, and loss of biomechanical function, accompanied by structural changes such as annulus fibrosus rupture, nucleus pulposus dehydration, and endplate calcification (Zhu et al., 2023; Xia et al., 2024). Current conventional clinical treatments (such as non-steroidal anti-inflammatory drugs, physical therapy, and surgical intervention), while capable of providing short-term symptomatic relief, cannot reverse the degenerative process or achieve tissue regeneration (Krut et al., 2021).

In recent years, regenerative medicine strategies centered on cell therapy have provided new directions for the fundamental treatment of IVDD. Among these, mesenchymal stem cells (MSCs) have become the most promising therapeutic tools due to their self-renewal, multilineage differentiation potential, low immunogenicity, and immunomodulatory properties (Ogaili et al., 2025). The basic strategy is to implant MSCs to promote the repair and functional reconstruction of degenerated tissue (Lu et al., 2021). Numerous studies have shown (Li et al., 2025; Tang et al., 2025; Zhao et al., 2025) that MSCs can not only directly differentiate into nucleus pulposus-like cells, replenishing the missing cell population (Li et al., 2025), but also play a key role through paracrine actions: on one hand, by secreting growth factors (such as TGF-β, IGF-1) to activate anabolic pathways and promote ECM synthesis (Tang et al., 2025); on the other hand, releasing anti-inflammatory factors (such as IL-10) and inhibiting the destructive effects of pro-inflammatory factors (such as IL-1β, TNF-α), thereby effectively improving the local microenvironment and delaying or even reversing the process of IVDD (Zhao et al., 2025). Notably, researchers have identified endogenous progenitor cells in multiple regions of the intervertebral disc, including the nucleus pulposus, annulus fibrosus, and endplate (Lyu et al., 2019; Hu et al., 2018). These cells not only express typical MSC surface markers but also have expression levels of pluripotency markers (such as OCT3/4, NANOG) that reach or even exceed those of MSCs, and possess trilineage (osteogenic, chondrogenic, adipogenic) differentiation capacity as well as self-renewal potential (Hu et al., 2018). These cells usually reside quiescently in specific stem cell niches within the intervertebral disc and can be activated under conditions of tissue injury or degeneration, differentiating into chondrocytes, osteoblasts, and adipocytes, indicating that the intervertebral disc possesses a certain self-repair potential, providing a new cell source for regenerative medicine (Shen et al., 2015).

However, as an avascular tissue, the intrinsic microenvironment of the intervertebral disc is characterized by extreme features such as hypoxia, low nutrient levels, high acidity, hyperosmolarity, and sustained mechanical stress (Zhang et al., 2022). During degeneration, this microenvironment further deteriorates, accompanied by the accumulation of inflammatory factors, increased protease activity, and abnormal biomechanical properties (Ikwuegbuenyi et al., 2025), severely affecting the survival, proliferation, differentiation, and functional expression of resident cells (including both endogenous cells and transplanted cells). Although some clinical studies have shown certain improvements from stem cell therapy (Munda and Velnar, 2024; Beall et al., 2020), the negative impact of the degenerative microenvironment on their biological behavior remains a major challenge, often leading to inconsistent efficacy or even treatment failure. Furthermore, different types of MSCs (such as bone marrow-derived, adipose-derived, disc-derived) exhibit significant differences in their response to microenvironmental stress (Wu et al., 2017; Xia et al., 2022), but the related mechanisms have not yet been systematically elucidated.

Therefore, when formulating regenerative treatment strategies, the adverse microenvironment of the degenerated intervertebral disc must be fully considered. With the progression of degeneration, the imbalance between catabolism and anabolism and the continuous deterioration of the microenvironment may further inhibit the activity of stem/progenitor cells, placing higher demands on treatment strategies. This article aims to systematically review the main characteristics and dynamic evolution of the intervertebral disc microenvironment under physiological and pathological conditions, focusing on analyzing the impact of the degenerative microenvironment on the biological behavior of exogenous MSCs and endogenous progenitor cells. It will also summarize current strategies to enhance MSC adaptability, survival rate, and functional expression, including genetic regulation, biomaterial-assisted transplantation, and microenvironment modulation, to provide a theoretical basis and feasible path for promoting the clinical translation of stem cell therapy in intervertebral disc regeneration.

## 2 Anatomy of the intervertebral disc and degeneration

The intervertebral disc (IVD) is a fibrocartilaginous structure located between adjacent vertebral bodies, composed of the outer annulus fibrosus (AF), the central nucleus pulposus (NP), and the superior and inferior cartilage endplates (CEP). It is the core structure for distributing mechanical loads, enabling bending movements, and providing shock absorption in the spine, with its good elasticity and osmotic properties providing flexibility and stability to the spine (Cassidy et al., 1989).

Anatomically, the annulus fibrosus tightly encircles the nucleus pulposus in a lamellar fashion. The collagen fiber type transitions gradually from the outer to the inner layers—the outer layer is primarily composed of tension-resistant type I collagen, while moving inward, it gradually mixes with type II collagen extending to the edge of the nucleus pulposus, forming a tough ring structure that provides mechanical support to the disc by restricting NP displacement and resisting compressive loads. The nucleus pulposus, as the functional core of the disc, is gel-like, rich in water (80%–90% of wet weight) and an ECM primarily composed of type II collagen and proteoglycans. Among these, proteoglycans adsorb large amounts of water through glycosaminoglycan side chains and form aggrecan complexes by binding with hyaluronic acid, creating a high osmotic pressure environment that maintains the highly hydrated state of the NP. This is a key component for ensuring disc height, distributing spinal physical loads, and facilitating the exchange of nutrients and metabolites (Adams and Green, 1993). The cartilage endplate, composed of hyaline cartilage, covers the upper and lower surfaces of the disc. It not only anchors the disc to the vertebral body and transmits vertical mechanical loads but also serves as a critical interface for disc metabolism, facilitating the exchange of nutrients and metabolic waste between the disc and the terminal vessels of the vertebral body through permeation. It is noteworthy that the entire intervertebral disc exists in a special microenvironment that is avascular, hypoxic (oxygen tension 5–150 mmHg), hyperosmotic (osmolarity 430–496 mOsm/L), and characterized by slow metabolism. Its function is highly dependent on the integrity of the ECM—the loss or destruction of the ECM will directly trigger intervertebral disc degeneration (IVDD) and structural changes (Zehra et al., 2015).

**FIGURE 1:**
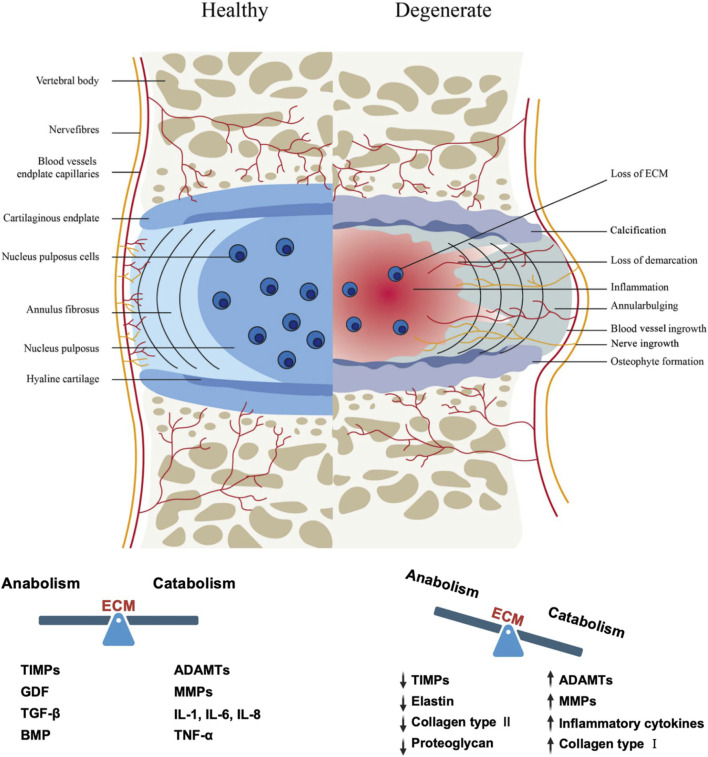
The intervertebral disc in healthy and pathological tissues (Lu et al., 2021). The healthy intervertebral disc (IVD) comprises three distinct regions: the gelatinous nucleus pulposus (NP), the annulus fibrosus (AF), and the cartilaginous endplate (CEP). Intervertebral disc degeneration (IVDD) is characterized by various structural changes including NP dehydration and inflammation, extracellular matrix metabolic imbalance, ingrowth of nerve fibers and blood vessels, annular bulging, and osteophyte formation at the CEP. TIMPs: Tissue Inhibitor of Metalloproteinases, MMPs: Matrix Metalloproteinases, ADAMTs: A Disintegrin and Metalloproteinase with Thrombospondin Motifs, GDF: Growth Differentiation Factor, TGF-β: Transforming Growth Factor-β, BMP: Bone Morphogenetic Protein, IL-1: Interleukin-1, IL-6: Interleukin-6, IL-8: Interleukin-8, TNF-α: Tumor Necrosis Factor-α.

The occurrence of IVDD results from the combined effects of genetic and environmental factors. Aging, smoking, infection, genetic predisposition, abnormal biomechanical loading (such as long-term bending or heavy lifting), and reduced nutrient supply through the cartilage endplate are all inducing factors (Wu et al., 2021). From a pathological evolution perspective, IVDD begins with the progressive destruction of the nucleus pulposus: the early stage is characterized by increased degradation of proteoglycans, leading to decreased water content in the NP and weakened biomechanical performance (reduced compressive load capacity), transferring more stress to the annulus fibrosus. The annulus fibrosus, having significantly weaker compression resistance than tensile resistance, gradually develops structural damage under continuous high pressure, manifesting as overall bulging beyond the endplate boundaries (visible as disc bulging on imaging). As the pathology progresses, the inner annulus fibrosus ruptures first, leading to localized protrusion of the nucleus pulposus (contained herniation); if the outer annulus fibrosus subsequently ruptures, the nucleus pulposus completely extrudes (non-contained herniation), potentially protruding into the spinal canal or lateral recess, compressing the cauda equina and nerve roots, causing pain or motor dysfunction (Buckwalter, 1995). At the microscopic level, IVDD manifests as deterioration of the disc microenvironment (hypoxia, low pH, inflammatory factor infiltration), a sharp reduction in cell number (necrosis, apoptosis, or pyroptosis), and imbalance in ECM metabolism (catabolism greater than anabolism). Macroscopically, it presents features such as annulus fibrosus deformation/rupture, nucleus pulposus water loss/fibrosis, cartilage endplate calcification, neovascularization and nerve ingrowth, vertebral osteophyte formation, and decreased disc height (Figure 1). Due to the lack of blood supply, once degeneration or injury occurs, the self-repair capacity of the disc is very poor, ultimately leading to clinical symptoms such as discogenic low back pain or sciatica associated with nerve compression.

## 3 Physiological characteristics and degenerative changes of the intervertebral disc microenvironment

The intervertebral disc is an avascular tissue, and its microenvironment has unique physiological characteristics. The distance from the blood supply can be up to 8 mm or more (Brodin, 1955). Nutrients can only slowly permeate from the capillaries in the adjacent vertebral body through the endplate (CEP) to the nucleus pulposus (NP), and the clearance of metabolic waste is also significantly restricted (Holm et al., 1981), resulting in cells existing in an extremely impoverished state.

Under normal physiological conditions, the center of the nucleus pulposus primarily relies on anaerobic glycolysis for metabolism, forming a nutrient gradient from the periphery to the center, characterized by a microenvironment with low glucose (approximately 1 mmol/L) and high lactate (approximately 5 mmol/L) (Mokhbi Soukane et al., 2009; Bartels et al., 1998). The pH of a healthy intervertebral disc ranges between 7.0 and 7.2 (Ichimura et al., 1991), about 0.5 units lower than the surrounding tissue fluid (Urban, 2002). The oxygen partial pressure ranges between 5 and 150 mm Hg, with an average oxygen concentration of about 2% (Bartels et al., 1998), significantly lower than most human tissues. The extracellular matrix (ECM) is primarily composed of type II collagen and aggrecan, the latter accounting for about 50% of the dry weight in the pediatric nucleus pulposus, endowing the tissue with high osmotic pressure (approximately 430–496 mOsm/L) (Ishihara et al., 1997; Urban and Roberts, 2003), and providing mechanical support, water retention capacity, and compressive resistance.

During degeneration, the intervertebral disc microenvironment deteriorates significantly. The endplate gradually calcifies and thins, further hindering substance exchange, leading to a drop in glucose concentration that can reach as low as half that of the healthy state (Holm et al., 1981; Jackson et al., 2011). The pH decreases to 5.7–6.5 due to proton accumulation (Kitano et al., 1993; Diamant et al., 1968), and oxygen tension drops to approximately 1% (Bartels et al., 1998). The degenerated disc presents a state of chronic inflammation, with upregulated expression of various inflammatory factors, including IL-1, TNF-α, MMPs (such as MMP10, MMP12), COX-2, IL-8, among others. (Peng and Lv, 2015; Lyu et al., 2021), among which IL-1 plays a central role in promoting matrix degradation and pain generation (Phillips et al., 2015). Simultaneously, the mechanical properties of the ECM change significantly: the shear stiffness of the human nucleus pulposus increases from 12.5 kPa in Pfirrmann grade 1–16.5 kPa in grade V (Walter et al., 2017), collagen fibers thicken and become disorganized (Leung et al., 2014), its aggrecan synthesis decreases while degradation increases (Antoniou et al., 1996), and osmotic pressure drops to about 300 mOsm/L (Wuertz et al., 2007) (Table 1).

**TABLE 1:** Characteristics of the intervertebral disc microenvironment under physiological and degenerative conditions.

Condition	Healthy IVD	IVDD
Vascularity	Avascular tissue with blood supply distances exceeding 8 mm; nutrients slowly permeate from adjacent vertebral body capillaries through the CEP to the NP, with significantly restricted metabolic waste clearance	Progressive calcification and thinning of the CEP further impede substance exchange, exacerbating nutrient supply difficulties
Hypoxia	Oxygen partial pressure ranges from 5 to 150 mmHg, with an average concentration of ∼2%; the central NP region can be as low as 0.65%	Oxygen tension further decreases to ∼1%, intensifying the hypoxic state
Low glucose concentration	Glucose concentration decreases from the boundaries towards the NP center, forming a nutrient gradient; central concentration is ∼1 mmol/L	Glucose concentration drops significantly, potentially to half that of healthy levels, worsening nutritional deprivation
Acidity	Due to anaerobic glycolysis and lactic acid production (∼5 mmol/L), average pH is 7.0–7.2, approximately 0.5 units lower than surrounding tissue fluid	The local pH drops to 5.6–6.5 due to nutrient depletion and lactic acid accumulation, forming a significantly acidic microenvironment
Hyperosmolarity	High aggrecan concentration confers tissue hyperosmolarity (430–496 mOsm/L)	Reduced synthesis and increased degradation of aggrecan lead to a drop in osmolarity to ∼300 mOsm/L
Mechanical loading	Mechanical stimuli(flexion, torsion, shear, and compression)regulate IVD cell activity and metabolism within a physiological range of 0.1–2.5 MPa	The loss of structural integrity in the IVDD precipitates pathological loading patterns throughout the spinal motion segment, ultimately resulting in tissue damage and cellular overstress
Inflammation	Pro-inflammatory cytokines and chemokines are potential contributors to key events in IVD development, including the recruitment of local progenitor cell populations	Elevated levels of proinflammatory cytokines promote a cascade of pathological events, including cellular apoptosis, senescence, autophagy, extracellular matrix breakdown, and ultimately, discogenic low back pain

CEP, cartilaginous end plate; NP, nucleus pulposus; IVD, intervertebral disc; IVDD, intervertebral disc degeneration.

In summary, intervertebral disc degeneration manifests as a comprehensive deterioration of the microenvironment, including increased ECM stiffness, reduced nutrients, increased acidity, hypoxia, and exacerbated inflammatory response. These changes collectively promote the progressive decline of disc structure and function.

## 4 Traditional treatments for IDD

Current treatments for intervertebral disc degeneration (IDD/IVDD) include conservative therapy and surgical intervention (Lu et al., 2021). Conservative treatment aims to relieve low back pain (LBP) and typically adopts a stepped analgesic strategy (Deyo and Weinstein, 2001): starting with non-steroidal anti-inflammatory drugs, potentially escalated to weak opioids if ineffective, and considering strong opioids only for severe, refractory pain. For patients with neuropathic pain components, antidepressants can be combined for synergistic analgesia and mood improvement. Local transdermal patches and epidural steroid injections can also provide temporary symptom relief for a certain period, but the latter, being an invasive procedure, carries associated risks and its efficacy is difficult to sustain. Conservative treatment is often combined with physical therapy. Surgical intervention is suitable for patients with severe symptoms, failure of conservative treatment, and significant limitations in daily function. Options include minimally invasive procedures (such as radiofrequency ablation) and open surgeries (such as discectomy, fusion, and arthroplasty, etc.) (Xin et al., 2022). Although surgery can improve function to some extent, it remains essentially a palliative measure that cannot reverse the degenerative process, and it may be accompanied by a series of intraoperative and postoperative risks, such as infection, bleeding, and implant failure.

Overall, existing clinical treatment methods primarily focus on symptom control and do not intervene in the fundamental pathological mechanisms of disc degeneration. Although many patients respond well in the short term, long-term outcomes remain unsatisfactory. In recent years, emerging strategies in regenerative medicine, such as stem cell therapy, dedicated to restoring disc structure and function at the cellular and molecular levels, have brought new hope for achieving disease-modifying therapies (Table 2).

**TABLE 2:** Comparison of stem cell therapy and traditional treatments.

Treatment method	Advantages	Indications	Limitations	Clinical application cases
Minimally invasive surgery	Minimal trauma, less bleeding, reduced pain Short hospital stay, rapid recovery Precise operation, minimizing damage to surrounding tissues	Contained disc herniation refractory to conservative treatment Spinal stenosis, lumbar spondylolisthesis, etc. Selected cases of adjacent segment disease (ASD)	Not suitable for severe, multi-level spinal degeneration Risk of complications like remnant nucleus, recurrence, or infection	Mature and commonly used techniques, such as UBE, endoscopic discectomy. Future involves continuous technological iteration and gradual expansion of indications
Open surgery	Provides definitive decompression of neural elements, with significant and durable effects Can reconstruct spinal stability	Severe neurological compression (e.g., severe spinal stenosis) Cervical disc herniation causing radiculopathy, unresponsive to conservative care Severe lumbar spondylolisthesis	Significant trauma and long recovery period Long-term risks such as adjacent segment disease (ASD)	Clinically well-established as the definitive solution for severe organic pathologies. Future focuses on integration with minimally invasive techniques and developing solutions for complications like ASD.
Open surgery	Provides definitive decompression of neural elements, with significant and durable effects Can reconstruct spinal stability	Severe neurological compression (e.g., severe spinal stenosis) Cervical disc herniation causing radiculopathy, unresponsive to conservative care Severe lumbar spondylolisthesis	Significant trauma and long recovery period Long-term risks such as adjacent segment disease (ASD)	Clinically well-established as the definitive solution for severe organic pathologies. Future focuses on integration with minimally invasive techniques and developing solutions for complications like ASD.
Stem cell therapy	Minimally invasive with the potential to repair or reverse degenerative structures, rather than merely alleviating symptoms Mediates tissue regeneration through dual mechanisms: cell differentiation and paracrine signaling Exerts immunomodulatory effects that suppress local inflammation and inhibit apoptosis	Primarily in the clinical research stage for chronic pain due to disc degeneration	Low cell survival rates attributable to the harsh IVD milieu (hypoxia, acidity) Concerns regarding potential tumorigenicity and unresolved ethical issues Absence of standardized treatment protocols and long-term safety profiles	Emerging as a promising direction with demonstrated potential for pain relief and functional recovery in early-stage trials The therapeutic prospect lies in its combination with biomaterials (e.g., hydrogels) or active herbal compounds to enhance cell delivery and viability
Stem cell + exosome therapy	Functions as critical paracrine mediators by enhancing stem cell viability and promoting reparative signaling Offers a cell-free therapeutic strategy with comparable regenerative potential to stem cells yet reduced risks Multi-targeted actions: balancing ECM anabolism/catabolism and mitigating inflammatory, and lleviating oxidative damage	In the pre-clinical research stage as a regenerative strategy to delay or repair disc degeneration	Major challenges in large-scale production and quality control Unsolved issues with targeted delivery efficiency in vivo Very limited clinical evidence	An emerging research direction, considered a promising future direction for cell-free regenerative therapy. Future lies in developing engineered exosomes (e.g., genetically modified) and smart delivery systems (e.g., hydrogels) to enhance efficacy

IVD: Intervertebral disc; Adjacent Segment Disease (ASD); ECM: extracellular matrix.

## 5 Stem cells in intervertebral disc repair

The most important pathological features of IVDD include metabolic imbalance of proteoglycans and type II collagen in the extracellular matrix (ECM), and a reduction in the number of nucleus pulposus cells (NPCs) (Kepler et al., 2013). Therefore, many therapeutic strategies aim to increase the number of NPCs, promote matrix metabolism and protein synthesis, thereby enhancing the mechanical load-bearing capacity of the disc and delaying or improving the process of IVDD (Xin et al., 2022). Stem cells, due to their high self-renewal capacity, multidifferentiation potential, wide range of sources, and ease of isolation and culture, show broad application prospects in regenerative medicine (Krut et al., 2021). In recent years, stem cell-based therapeutic strategies have made significant progress in IDD, providing new avenues for restoring disc structure and function by differentiating into nucleus pulposus-like cells and promoting ECM synthesis.

Previous studies have shown (Li et al., 2025; Xia et al., 2022; Haufe and Mork, 2006) that stem cells participate in IVDD repair through multiple mechanisms: they can differentiate into nucleus pulposus cells, annulus fibrosus cells, etc., in a suitable microenvironment, and regulate differentiation and ECM synthesis through signaling pathways such as TGF-β and Wnt/β-catenin (Li et al., 2025); they exert paracrine actions, secreting various cytokines and exosomes to modulate local inflammation, inhibit cell apoptosis, and promote ECM regeneration (Xia et al., 2022); they also directly participate in the structural reconstruction of the ECM, improving the elasticity and height of the intervertebral disc (Haufe and Mork, 2006). Notably, since transplanting stem cells into the degenerative intervertebral disc (IVD) is an invasive procedure, research focus is gradually shifting towards the possibility of utilizing exogenous or endogenous MSC homing to the IVD (Croft et al., 2021). MSC homing refers to the process by which cells migrate from their original niche to injured or pathological tissue, a process mediated by various growth factors and chemokines (Wangler et al., 2019). However, endogenous repair strategies for IDD are still in the preclinical research stage and require further in-depth study.

**FIGURE 2:**
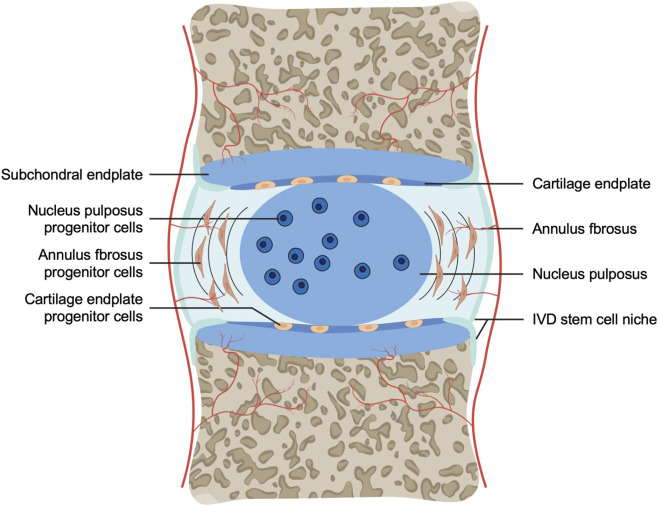
IVD progenitor cells and the stem cell niche. Progenitor cells have been identified in the nucleus pulposus, annulus fibrosus, and cartilaginous endplates of the intervertebral disc, and its stem cell niche is suggested to be located in the perichondrium adjacent to the epiphyseal plate and the outer annulus fibrosus.

Based on cell source, stem cells used for the biological treatment of IDD can be divided into two major categories: endogenous and exogenous. The first category is intervertebral disc endogenous stem cells, residing located within the disc. Under ideal conditions, tissue repair or reversal of degeneration could be achieved by activating and inducing their proliferation and differentiation (Hu et al., 2019). Intervertebral disc tissue-specific stem/progenitor cells (IVDSPCs) and their residing “niche” structure are distributed in specific anatomical regions, typically in a quiescent state and can be activated by signals such as tissue injury to participate in repair (Lyu et al., 2019). This niche is a specific structure that maintains stem cell quiescence, composed of a special extracellular matrix and neighboring cells, precisely regulating stem cell proliferation and differentiation (Gao et al., 2022) (Figure 2). Henriksson et al. (2009) first identified niches in the annulus fibrosus-ligament junction zone and perichondrium in a rabbit model using BrdU labeling, where slow-cycling cells expressed β1 integrin and epithelial-mesenchymal transition-related transcription factors SNAI1/SNAI2. By anatomical location, IVDSPCs can be divided into nucleus pulposus-derived progenitor cells (NPSPCs), annulus fibrosus-derived progenitor cells (AFSPCs), and cartilage endplate-derived progenitor cells (CESPCs) (Hu et al., 2019). Although progenitor cells from different regions of the disc exist, recent research suggests they may share a common biological origin and characteristics. Multiple studies have confirmed their existence and differentiation potential: Risbud et al. (2007) identified a cell population with trilineage differentiation capacity in the human annulus fibrosus; Sakai et al. (2012) discovered Tie2-positive (Tie2+) and disialoganglioside 2-positive (GD2+) progenitor cell populations in the nucleus pulposus of mice and humans, which formed spheroid colonies expressing type II collagen and aggrecan. Furthermore, Kim et al. (2003) proposed that rabbit notochordal cells could promote the migration of cartilage endplate cells to the nucleus pulposus, driving the transition from notochord to a fibrocartilaginous phenotype; the Liu et al. (2017) and Xiong et al. (2012) also isolated multipotent CEPSCs from the human cartilage endplate and found their migration ability regulated by specific factors.

Additionally, IVDSPCs share similar surface markers with bone marrow mesenchymal stem cells (MSCs), expressing CD105, CD73, and CD90, while not expressing hematopoietic lineage markers such as CD14, CD34, CD45, or HLA-DR (Kim et al., 2003). They may also express stemness markers such as SOX2, OCT3/4, NANOG, CD133, and nestin, possessing osteogenic, adipogenic, and chondrogenic differentiation capabilities (Xiong et al., 2012). Notably, Sakai et al. (2012) proposed that Tie2+ and GD2+ are specific surface markers for NPSPCs, providing a new basis for distinguishing IVDSPCs from ordinary disc cells.

The second category is exogenous mesenchymal stem cells (MSCs), isolated from other tissues in the body, with a wide range of sources, including bone marrow, adipose tissue, umbilical cord blood, etc. Common types include umbilical cord mesenchymal stem cells (UCMSCs), bone marrow mesenchymal stem cells (BMSCs), and adipose-derived stem cells (ADSCs), as well as less common like umbilical cord blood-derived MSCs (UCB-MSCs), and synovial or peripheral blood-derived MSCs (Zhang et al., 2022). These MSCs each have advantages and limitations in treating IDD (Table 3). Exogenous MSCs typically possess strong proliferation and multidifferentiation capacity, low immunogenicity, and anti-inflammatory functions, and can regulate nucleus pulposus cell behavior through direct contact, indirect signaling, or non-contact co-culture (Lu et al., 2021). Currently, MSCs are the most commonly used stem cell type in IVDD treatment, with BMSCs being the most widely studied.

**TABLE 3:** Summary of the characteristics of stem cells from different sources.

Stem cell source	Types	Advantages	Disadvantages
Intervertebral disc-derived stem cells	Nucleus pulposus stem cells (NPSCs) Annulus fibrosus stem cells (AFSCs) Cartilaginous endplate stem cells (CESCs)	Exhibits enhanced compatibility with the demanding microenvironment of the intervertebral disc	Lack of standard, reliable and efficient separation and purification methods
Mesenchymal stem cells (MSCs)	Bone marrow mesenchymal stem cells (BM-MSCs)	High chondrogenic differentiation potential	The acquisition process is cumbersome and traumatic, and the density of MSCs in bone marrow aspirate is low
	Adipose-derived mesenchymal stem cells (AD-MSCs)	Easy to obtain in large quantities, low morbidity at the donor site and high proliferation rate	Chondrogenic differentiation potential is not as good as that of BM-MSCs
	Umbilical cord mesenchymal stem cells (HUC-MSCs)	Easy to obtain, low immunogenicity, suitable for use in allogeneic stem cell transplantation	Ethical issues, insufficient differentiation potential
Pluripotent stem cells (PSCs)	Embryonic stem cells (ESCs)	Can differentiate into all cell types of the three germ layers	Ethical issues, possible immune rejection after transplantation, risk of teratoma formation
	Induced pluripotent stem cells (iPSCs)	Can differentiate into all cell types of the three germ layers, less immune rejection and ethical issues	Risk of teratoma formation

## 6 The impact of the disc microenvironment on MSCs and disc progenitor cells

**FIGURE 3:**
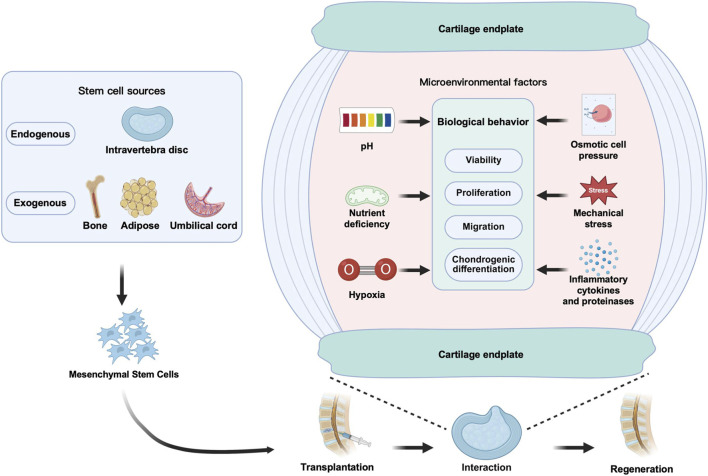
Schematic diagram of the effects of six IVD microenvironmental factors on transplanted MSCs.

As the “soil” upon which “seed” cells depend for survival, the impact of the microenvironment on the function of mesenchymal stem cells in disease states and during treatment is increasingly garnering attention. Although exogenous stem/progenitor cell transplantation is considered a potential strategy for treating intervertebral disc degeneration (IVDD), its efficacy is severely constrained by the aforementioned microenvironment. It is noteworthy that different types of stem/progenitor cells exhibit significant differences in their response and tolerance to various microenvironmental stress factors. Systematically analyzing the impact of these microenvironmental factors on MSCs and their mechanisms will help in developing effective strategies to promote endogenous MSC-targeted repair and exogenous MSC-mediated disc regeneration. The following sections will systematically elaborate on the specific effects of different microenvironmental factors on transplanted stem cells (Figure 3).

### 6.1 Avascularity and nutrient deficiency

The intervertebral disc (IVD) is the largest avascular tissue in the human body, and its cells depend on the diffusion of nutrients from surrounding blood vessels through the cartilage endplate (CEP) into the tissue interior. Glucose, as a key substrate for energy metabolism, exhibits a concentration gradient in the normal IVD, decreasing from about 5 mM in the outer annulus fibrosus (AF) to about 1 mM in the central nucleus pulposus (NP) (Shirazi-Adl et al., 2010). After IVD degeneration (IDD), due to CEP calcification and reduced vertebral blood supply, the glucose concentration in the NP further decreases, with an average concentration in degenerated human NP being only 0.603 ± 0.108 mM (Yin et al., 2020), approaching the minimum threshold required for IVD cell survival (0.5 mM) (Guehring et al., 2009). Besides glucose, growth factors and proteins contained in serum are also important nutritional sources for maintaining cell function.

Under low glucose conditions, mesenchymal stem cells (MSCs) exhibit certain adaptive responses. Studies (Wuertz et al., 2008; Liang et al., 2012) show that an IVD-like low glucose environment (approximately 1–5.5 mM) mildly inhibits cell viability but can promote the expression of chondrogenic differentiation-related genes like aggrecan and slightly enhance proliferation. For example, rat bone marrow MSCs (BMSCs) cultured in 1 mg/mL glucose produced higher yields of aggrecan and collagen I in the extracellular matrix compared to the high glucose group (Wuertz et al., 2008); human adipose-derived MSCs (AD-MSCs) in low glucose had viability reduced to 73%, but aggrecan production increased to 170% (Liang et al., 2012). In contrast, very low glucose (e.g., 1 mM or 0.25 mg/mL) significantly inhibits cell viability, proliferation, and glycosaminoglycan synthesis, and promotes apoptosis (Naqvi and Buckley, 2015; Stolzing et al., 2006). Notably, NP-derived MSCs (NP-MSCs) also show inhibited proliferation and increased apoptosis under severe nutrient deprivation (Junger et al., 2009; Tian et al., 2020). Serum deprivation has a more pronounced effect on MSCs. Serum-free conditions generally inhibit cell viability, proliferation, and multipotency, but can enhance chondrogenic differentiation potential (Parker et al., 2007; Wan et al., 2016; Pang et al., 2021). AD-MSCs maintain relatively good survival rates and chondrogenic capacity under serum deprivation and show higher tolerance compared to BMSCs (Takahashi et al., 2018). However, serum deprivation can also induce apoptosis, particularly evident in BMSCs and CEP progenitor cells (Zhu et al., 2006; Fu et al., 2015; He et al., 2017). Interestingly, NP progenitor cells under serum-free conditions only show transient morphological changes (semi-adherent morphology), and most survive after serum restoration, suggesting low serum dependence (Turner et al., 2016).

Under physiological states, hypoxia often coexists with nutrient deficiency in the degenerated intervertebral disc (IVD), forming a complex stress microenvironment. Oxygen-glucose deprivation (OGD) or its combination with serum deprivation (OGSD) can significantly inhibit the viability, proliferation, migration, and multidifferentiation capacity of mesenchymal stem cells (MSCs), and induce oxidative stress and apoptosis (Turner et al., 2016; Ghorbani et al., 2018; Abdolmaleki et al., 2020; Pan et al., 2022; Li C. et al., 2018; Yang et al., 2022). For example, in AD-MSCs, OGD treatment not only inhibits adipogenic differentiation but also reduces their migration capacity (Yang et al., 2022); in NP-MSCs, the combined effect of hypoxia and nutrient deprivation inhibits cell proliferation, induces apoptosis, enhances caspase-3 activity, and downregulates functional genes (e.g., proteoglycan, collagen-I and -II) and stem cell-related genes (including Oct4, Nanog, Jagged, and Notch1) (Tian et al., 2020). Additionally, some studies have focused on the effects of hypoxia and serum deprivation on rare types of MSCs. Huang YC. et al. (2009), Huang et al. (2010) reported that hypoxia significantly promoted the proliferation of placenta-derived MSCs (PMSCs), while serum deprivation inhibited their growth. Notably, in PMSCs, combined hypoxia and serum deprivation did not significantly induce apoptosis, a phenomenon potentially related to the upregulation of BCL-2 expression under serum deprivation conditions (Huang YC. et al., 2009). These results suggest that PMSCs have certain application potential in ischemia-related tissue engineering. To enhance MSC adaptability in ischemic microenvironments, various protective strategies have been proposed. Small molecule compounds like berberine reduce autophagy and apoptosis by activating the AMPK signaling pathway (Pang et al., 2021; Ghorbani et al., 2018); acetyl-L-carnitine (ALC) can regulate the balance of survival/death signals (Abdolmaleki et al., 2020; Pan et al., 2022); exogenous factors like TGF-β3 inhibit apoptosis by promoting DNA repair (Wu F. et al., 2018; Li et al., 2019), and IGF-1 alleviates functional inhibition of NP-MSCs via the PI3K pathway (Tian et al., 2020). Furthermore, inhibiting RIP3 can improve the viability and migration of AD-MSCs under OGD and reduce inflammatory responses (Yang et al., 2022).

Notably, moderate nutritional stress may also have positive significance for maintaining stem cell stemness. Serum starvation can induce NP-MSCs to enter a quiescent state, which is conducive to stemness maintenance (Li et al., 2019); low glucose environments enhance the expression of pluripotency markers (e.g., SOX-2, OCT-4) in AD-MSCs, although high glucose generally promotes their senescence and reduces stemness (Stolzing et al., 2006; Chen et al., 2016; Stolzing et al., 2010; Stolzing et al., 2012; Liu et al., 2020). These seemingly contradictory results suggest that glucose concentration and exposure duration have complex effects on MSC behavior, requiring further analysis in conjunction with *in vivo* microenvironment characteristics.

In summary, IVD-like low glucose and serum-deficient environments, while posing challenges to MSC survival, may promote their chondrogenic differentiation and maintain some stemness. However, severe nutrient deprivation or combination with hypoxia will significantly impair cell function. Enhancing MSC stress adaptability through pharmacological or factor interventions may provide new strategies for improving the efficacy of cell transplantation therapy for IDD.

### 6.2 Hypoxia

In the normal adult intervertebral disc (IVD), oxygen partial pressure gradually decreases from about 10% in the outer annulus fibrosus to less than 1% in the central region, placing the entire disc in a state of continuous hypoxia, which may further decrease during degeneration (Urban, 2002). Although mesenchymal stem cells (MSCs) are typically assessed for their therapeutic potential under normoxic conditions, they will be subjected to severe hypoxia after transplantation.

Studies indicate that hypoxia can promote the viability, proliferation, and migration of bone marrow-derived MSCs (BMSCs) (Grayson et al., 2006; Grayson et al., 2007; Tsai et al., 2011; Wang W. et al., 2018), and inhibit senescence and maintain stemness by downregulating E2A–p21 via the HIF-1α–Twist pathway to upregulate telomerase activity (Grayson et al., 2007; Tsai et al., 2011); however, some studies also report that hypoxia may inhibit BMSC migration through HIF-1α and RhoA pathways (Fotia et al., 2015). Furthermore, hypoxia can alleviate the interleukin-1β (IL-1β)-induced inhibition of BMSC chondrogenic differentiation (Kanichai et al., 2008), improve the quality of extracellular matrix (ECM) synthesis (Risbud et al., 2004; Felka et al., 2009), and promote their differentiation towards a nucleus pulposus (NP)-like phenotype (Muller et al., 2011; Stoyanov et al., 2011), with HIF-1α being a key molecule mediating this chondrogenic promotion effect (Kanichai et al., 2008). These findings support the use of hypoxic preconditioning to enhance the ability of BMSCs to repair degenerated discs (Wang W. et al., 2018; Peck et al., 2021).

For adipose-derived MSCs (AD-MSCs), hypoxia can promote migration (Hwang et al., 2020) and chondrogenic differentiation, but results regarding cell viability and proliferation are controversial (Wan et al., 2016; Merceron et al., 2010; Schiller et al., 2013). Most studies show that hypoxia enhances their viability and proliferation (Fotia et al., 2015; Hwang et al., 2020; Jurgens et al., 2012; Wan et al., 2017), and their tolerance is better than that of BMSCs (Takahashi et al., 2018); however, some studies show that hypoxia inhibits the viability and proliferation of AD-MSCs and NP-MSCs while promoting chondrogenic differentiation (Li et al., 2013; Chung et al., 2012), or enhances mechanical resistance by activating autophagy (He et al., 2021). Additionally, hypoxia can also promote the proliferation and migration of umbilical cord mesenchymal stem cells (UC-MSCs) (Lee et al., 2015). The aforementioned controversies may stem from differences in experimental protocols, culture medium composition, oxygen tension levels, and donor heterogeneity, requiring further research for clarification.

Other MSCs such as placenta-derived (PMSCs), annulus fibrosus-derived (AF-MSCs), and peripheral blood-derived (PB-MSCs) also show enhanced proliferation, migration, or stemness under hypoxia (Ni et al., 2014; Choi et al., 2016; Li et al., 2017; Kwon et al., 2017; Casciaro et al., 2020; Wang P. et al., 2022), while synovial MSCs (SMSCs) exhibit enhanced chondrogenic differentiation capacity (Silva et al., 2020).

Notably, MSCs exhibit strong tolerance to hypoxic environments: Huang S. et al. (2013) found that monkey BMSCs cultured in 3.5% O2 for 10 days did not undergo apoptosis; human AD-MSCs in 1.5%–2% O2 had lower apoptosis rates (Wan et al., 2017; Feng et al., 2015). Short-term hypoxia (2%–5% O2, 48 h) can enhance the proliferation of human and porcine BMSCs (Antebi et al., 2018), although medium-term culture may temporarily slow the proliferation of rat AD-MSCs (Li et al., 2013) and human BM-MSCs (Grayson et al., 2006; Antebi et al., 2018), the total cell number can recover to normoxic levels after long-term culture (30 days), and differentiation capacity is maintained even after multiple passages (Grayson et al., 2007). Furthermore, hypoxia also enhances the expression of stemness markers (Oct4, Rex1) and colony formation without inducing tumorigenicity (Grayson et al., 2006; Tsai et al., 2011; Fotia et al., 2015; Choi et al., 2014). Regarding differentiation, hypoxia promotes chondrogenesis in rat, porcine BM-MSCs, and rat AT-MSCs (Kanichai et al., 2008; Li et al., 2013; Meyer et al., 2010), but its effects on osteogenic and adipogenic differentiation are inconsistent: some studies show increased osteogenic/adipogenic marker expression (Grayson et al., 2006; Valorani et al., 2012; Yu et al., 2018), while others report inhibition (Wan et al., 2016; Merceron et al., 2010; Schiller et al., 2013; Choi et al., 2014; D'Ippolito et al., 2006; Potier et al., 2007). Additionally, hypoxia enhances the anti-inflammatory capacity of BM-MSCs, such as inhibiting IL-8 and increasing IL-1ra and GM-CSF expression (Antebi et al., 2018). In contrast, disc progenitor cells under short-term hypoxia may have stimulated proliferation, but long-term hypoxia leads to their apoptosis (Li et al., 2013; Huang S. et al., 2013); also, similar to MSCs, the differentiation potential of disc progenitor cells is affected by hypoxia: hypoxia promotes chondrogenic differentiation of NP progenitor cells and inhibits osteogenic differentiation of human endplate (CEP) progenitor cells (Li et al., 2013; Yao et al., 2017; Yao et al., 2016).

In summary, hypoxia regulates MSC survival, proliferation, migration, stemness maintenance, differentiation direction, and anti-inflammatory function through the HIF-1α/HIF-2α signaling pathway, playing a positive role in disc repair. Although current results show some heterogeneity, hypoxic preconditioning remains a potential strategy to improve stem cell transplantation adaptability. Particularly for disc-derived stem cells (e.g., NP-MSCs), moderate hypoxia (e.g., 2% O2) may be beneficial for maintaining their stemness and bioactivity, but further research is needed to clarify the optimal conditions and mechanisms.

### 6.3 pH

In the avascular environment of the intervertebral disc (IVD), energy metabolism primarily relies on glycolysis, producing large amounts of lactic acid, placing the healthy nucleus pulposus (NP) in an acidic environment (pH 7.0–7.2). With the development of intervertebral disc degeneration (IDD), the pH can drop to 6.5 or even 5.6–5.7 (Kitano et al., 1993; Diamant et al., 1968), severely affecting the survival and function of endogenous or transplanted cells.

Acidic conditions significantly inhibit the viability, proliferation, and extracellular matrix (ECM) synthesis of mesenchymal stem cells (MSCs) (Diamant et al., 1968). Studies show that at pH ≤ 6.8, the expression of aggrecan and collagen II in BMSCs and AD-MSCs decreases, and cell viability and proliferation are impaired (Wuertz et al., 2008; Liang et al., 2012; Li et al., 2012), but changes in collagen I expression are inconsistent (Wuertz et al., 2008; Liang et al., 2012). In contrast, nucleus pulposus-derived MSCs (NP-MSCs) exhibit stronger acid tolerance, with less inhibition of their activity, proliferation, and matrix synthesis in acidic environments (Liu et al., 2017; Han et al., 2014).

Acid-sensing ion channels (ASICs), particularly ASIC1a and ASIC3, play a key role in this process (Swain et al., 2012; Ding et al., 2021). ASICs are a class of cation channels activated by extracellular protons (Yermolaieva et al., 2004), among which ASIC1a and ASIC3 are expressed in both MSCs and NP-MSCs (Ding et al., 2021; Li et al., 2014a; Cuesta et al., 2014). Acidic activation of ASICs causes Ca^2+^ influx, triggering mitochondrial pathway apoptosis (Ding et al., 2021; Cai et al., 2019). Inhibiting ASICs or the Notch signaling pathway (e.g., using Sa12b peptide) can enhance the survival ability of NP-MSCs under low pH (Wang Z. et al., 2022). TGF-β3 pretreatment can also improve the survival and matrix accumulation of BMSCs in acidic environments (Gansau and Buckley, 2021).

Notably, the duration and degree of acidic exposure may have dual effects: short-term acidic treatment (pH 6.5–6.8, 1–7 days) can enhance the stemness of BMSCs, promoting spheroid colony formation (Massa et al., 2017); but long-term exposure significantly impairs osteogenic differentiation capacity and inhibits ECM accumulation (Wuertz et al., 2008; Massa et al., 2017). Furthermore, lactate, as the main metabolic product of the acidic microenvironment, has concentration-dependent effects: low concentrations (5–10 mmol) may have anti-inflammatory and anti-apoptotic effects, while high concentrations (≥15 mmol) promote inflammatory factor expression and cell apoptosis (Schneider et al., 2012). This suggests that lactate accumulation in severely degenerated discs may further impair cell function.

In summary, the acidic microenvironment hinders the regenerative effects of MSCs and disc progenitor cells by inhibiting cell activity, proliferation, and ECM synthesis, with NP-MSCs exhibiting relatively stronger adaptability. Interventions targeting ASICs and cell preconditioning strategies may help improve the efficacy of cell therapy in low pH environments.

### 6.4 Osmotic pressure

In intervertebral disc (IVD) tissue, the normal extracellular osmotic pressure is maintained at a high level (430–496 mOsm/L), primarily due to the hyperosmotic environment created by the ECM rich in negatively charged aggrecan (Liang et al., 2012; van Dijk et al., 2011; Johnson et al., 2014; Lai et al., 2000). During disc degeneration, osmotic pressure gradually decreases due to proteoglycan loss, potentially dropping to about 300 mOsm in severe cases (Ishihara et al., 1997; Sadowska et al., 2018).

Multiple studies indicate that hyperosmotic environments inhibit the viability, proliferation, and ECM synthesis of various stem cells. Wuertz et al. (2008) and Liang et al. (2012) found that compared to standard culture conditions (280 mOsm), IVD-level hyperosmolarity (485 mOsm) significantly inhibited the cell viability, proliferation, and expression of aggrecan and collagen I in BMSCs and AD-MSCs. Li H. et al. (2018) cultured NP-MSCs under osmotic pressures simulating different degrees of degeneration (300–500 mOsm) and found that hyperosmotic environments (430 and 500 mOsm) inhibited their proliferation and chondrogenic differentiation capacity, while mild hypo-osmolarity (400 mOsm) was beneficial for cell proliferation and differentiation. Tao et al. (2013) also reported that hyperosmolarity inhibited the viability, proliferation, and expression of SOX-9, aggrecan, and collagen II in NP-MSCs and NPCs, but NP-MSCs exhibited stronger tolerance than NPCs. Furthermore, the culture model significantly influences the cellular response in hyperosmotic environments: AD-MSCs showed stronger tolerance in monolayer culture, but their tolerance to hyperosmotic environments was significantly reduced in 3D suspension or hydrogel cultures (Potocar et al., 2016).

Regarding chondrogenic/NP-like differentiation, existing research results show some divergence. Most studies report that hyperosmolarity inhibits differentiation-related gene and protein expression (Tao et al., 2013; Potocar et al., 2016); however, some studies indicate that moderate hyperosmolarity (around 400 mOsm) may promote the differentiation of AD-MSCs and BMSCs towards a chondrogenic phenotype and upregulate markers such as collagen II and aggrecan (Caron et al., 2013; Ahmadyan et al., 2018). Zhang Y. et al. (2020) further proposed the existence of an “optimal osmotic pressure window,” where 400 mOsm promotes NP-like differentiation of AD-MSCs, while 300 or 500 mOsm produces inhibitory effects. NFAT5 (TonEBP), as a key osmosensing transcription factor, participates in differentiation regulation by modulating Sox9 (Caron et al., 2013). Ahmadyan et al. (2018) compared the effects of different osmolytes (NaCl, sorbitol, PEG) and found that hyperosmolarity generally enhanced chondrogenesis, with NaCl potentially promoting hypertrophy, while sorbitol and PEG had superior effects and inhibited hypertrophy, with NFAT5 playing a key role in different osmotic adaptations. Additionally, mechanistically, hyperosmolarity can trigger cell cycle arrest (G1/G2 phase), reduce DNA synthesis, activate the p38 MAPK and ATM–p53–p21 signaling pathways, and may also induce a DNA damage response (Mavrogonatou and Kletsas, 2009).

In summary, the IVD hyperosmotic environment typically inhibits cell growth and anabolism, but a moderate increase in osmotic pressure may induce chondrogenic/NP-like differentiation. The decrease in osmotic pressure due to degeneration may be more conducive to cell proliferation and function, with the NFAT5 signaling pathway playing a key role in mediating osmotic pressure response and differentiation.

### 6.5 Mechanical stress

In intervertebral disc (IVD) tissue, cells are continuously subjected to various mechanical stresses including compression, tension, shear, torsion, and hydrostatic pressure. The intensity, frequency, duration, and direction of these forces significantly influence the metabolic behavior and fate determination of IVD cells and implanted stem cells (Xiang et al., 2024; Gao X. et al., 2016). Generally, dynamic loading (e.g., cyclic compression) often promotes anabolism, while static loading mostly leads to catabolic responses and cell dysfunction (Dudek et al., 2023; Liu et al., 2024; Gullbrand et al., 2015). These mechanical environments not only regulate the metabolism of the disc tissue itself but also profoundly affect the biological behavior of implanted mesenchymal stem cells (MSCs).

Cyclic compressive loading can promote the differentiation of MSCs towards a chondrogenic/NP-like phenotype. Studies show that such loading can upregulate TGF-β expression and enhance chondrogenesis in BMSCs (Huang et al., 2005; Huang et al., 2004; Mouw et al., 2007). For AD-MSCs, low-magnitude (5%) dynamic compression promotes ECM synthesis by inhibiting the TRPV4 channel without affecting cell viability; while higher magnitudes produce inhibitory effects (Gan et al., 2018). Dynamic compression can also produce additive effects when combined with exogenous Sox9, further promoting chondrogenic differentiation (Zhang et al., 2015). Similarly, cyclic hydrostatic pressure enhances the chondrogenic differentiation of BMSCs (Angele et al., 2003; Miyanishi et al., 2006) and AD-MSCs (Ogawa et al., 2009) in a dose- and time-dependent manner, and shows synergistic effects when co-cultured with nucleus pulposus cells (Dai et al., 2014). The molecular mechanism involves regulation of the Wnt signaling pathway: 120 kPa hydrostatic pressure can activate both the canonical Wnt/β-catenin and non-canonical Wnt/Ca2+ pathways (Cheng et al., 2022). However, excessively high stress (e.g., 1.0 MPa hydrostatic pressure) can induce apoptosis in NP-MSCs, but hypoxic conditions can mitigate this damage (Jiang et al., 2018; Li Z. et al., 2018).

In contrast, static loading generally produces negative effects. Static compression significantly inhibits the viability, migration, clonogenicity, and multidifferentiation potential of NP-MSCs and reduces their stemness, suggesting it may play an important role in hindering endogenous disc repair (Liang et al., 2018). Abnormally high compressive stress also leads to decreased mitochondrial membrane potential, ATP depletion, and reactive oxygen species accumulation in NP-MSCs, ultimately promoting apoptosis (Li Z. et al., 2018; Huang et al., 2020). Besides compressive loads, cyclic tensile strain (e.g., 10%, 1 Hz) can specifically promote collagen I synthesis in BMSCs, inducing fibrocartilage-like differentiation, but has no significant effect on collagen II and aggrecan (Connelly et al., 2010; Baker et al., 2011). Co-culturing AD-MSCs with articular chondrocytes at a 1:3 ratio combined with tensile stimulation significantly enhanced cartilage ECM production (Abusharkh et al., 2021).

Furthermore, the physical properties of the substrate (such as stiffness and fiber alignment) also significantly regulate stem cell differentiation. Softer substrates promote chondrogenesis in MSCs and NP progenitor cells, upregulating aggrecan, Sox9, and collagen II expression (Olivares-Navarrete et al., 2017; Park et al., 2011a; Navaro et al., 2015); whereas stiffer substrates promote osteogenic differentiation (Navaro et al., 2015; Li et al., 2014b; Lin et al., 2018). Scaffolds with aligned fibers can enhance the expression of collagen I and aggrecan in AF progenitor cells (Liu et al., 2015). Increased material elasticity stimulates collagen I expression but inhibits collagen II and aggrecan (Zhu et al., 2016), indicating that the mechanical properties of the substrate are important regulators of directed differentiation.

In summary, the effects of mechanical stress on MSCs and intervertebral disc-derived stem cells exhibit dependency on loading mode, specific parameters, and microenvironmental context. Dynamic and moderate mechanical stimulation generally promotes chondrogenic differentiation and matrix synthesis, whereas static or excessive loading tends to inhibit cellular functions and induce apoptosis. These findings provide a critical biomechanical rationale for optimizing tissue engineering strategies and regenerative therapies.

### 6.6 Inflammatory cytokines and proteinases

During intervertebral disc degeneration (IDD), the formation of an inflammatory microenvironment is a core pathological feature (Chen et al., 2023). The healthy nucleus pulposus (NP) tissue is in an immune-privileged state due to being encapsulated by the annulus fibrosus (AF) and cartilage endplate (CEP) (Zheng et al., 2024), but when the AF is damaged, NP antigens are exposed, activating an autoimmune response, leading to infiltration of immune cells such as macrophages and T lymphocytes, and the release of numerous inflammatory mediators (Wang et al., 2024; Xu et al., 2024), thereby accelerating IDD progression.

The inflammatory milieu within the degenerated intervertebral disc (IVD) not only drives tissue degeneration but also critically modulates the fate and function of therapeutic mesenchymal stem cells (MSCs). Key pro-inflammatory cytokines, including TNF-α, IL-1β, IL-6, and IL-17, are significantly upregulated in the degenerative nucleus pulposus (NP) (Risbud and Shapiro, 2014). They promote the expression of catabolic enzymes (e.g., MMP-1, -2, -3, -13, and ADAMTS-4/5) and inhibit anabolic proteins like aggrecan and collagen II by activating signaling pathways such as NF-κB, MAPK, and Toll-like receptors (TLR), ultimately leading to ECM degradation and tissue destruction (Lyu et al., 2021; Yang et al., 2025; Zhang GZ. et al., 2021). Simultaneously, anti-inflammatory cytokines such as TGF-β, GDF-5, GDF-6, IL-4, and IL-10 are also present in the degenerative environment and can, to some extent, delay degeneration and alleviate pain (Wang et al., 2016), with their balance potentially closely related to the polarization state of macrophages (Yamamoto et al., 2022; Djuric et al., 2020).

These inflammatory factors significantly influence the biological behavior of MSCs in a manner that is both concentration- and cell source-dependent. For example, IL-1β promotes BMSC migration via the NF-κB pathway but inhibits their chondrogenic differentiation—an effect that can be alleviated under hypoxic conditions (Felka et al., 2009; Carrero et al., 2012; Teixeira et al., 2018). Low concentrations of TNF-α (0.1–10 ng/mL) promote the proliferation and migration of NP-MSCs but inhibit their differentiation, whereas high concentrations (50–200 ng/mL) induce apoptosis (Cheng et al., 2019). IL-6 inhibits BMSC chondrogenic differentiation and limits proliferation via the JAK/STAT pathway (Yang et al., 2018; Kasprzycka et al., 2019; Huang H. et al., 2009), while IL-17 promotes BMSC proliferation, migration, and osteogenic differentiation through a process involving ROS and the p38/ERK-MAPK signaling pathway (Huang H. et al., 2009; Mojsilovic et al., 2011). Notably, IL-17 pretreatment enhances the homing ability and immunomodulatory function of BMSCs, suggesting potential therapeutic value (Ma et al., 2018). On the other hand, anti-inflammatory factors also play key regulatory roles. Members of the TGF-β superfamily (TGF-β1/2/3, GDF-5, and GDF-6) can all promote MSC differentiation towards NP-like cells, with GDF-6 having a particularly significant effect, inducing richer proteoglycan ECM synthesis, and showing more pronounced effects in AD-MSCs compared to BMSCs (Stoyanov et al., 2011; Tao et al., 2015; Clarke et al., 2014; Colombier et al., 2016; Gantenbein-Ritter et al., 2011). Mechanistically, TGF-β3 promotes chondrogenic differentiation via the Wnt5a/β-catenin signaling pathway (Jin et al., 2006; Li et al., 2020). IL-4, another important anti-inflammatory cytokine *in vivo*, improves MSC migration upon pretreatment, thereby positively influencing their regenerative function (Kasprzycka et al., 2019; Archacka et al., 2020; Zimowska et al., 2020).

Interestingly, different types of MSCs exhibit distinct responses to inflammatory stimuli. Notably, in adipose-derived MSCs (AD-MSCs), high concentrations of IL-1β and TNF-α promote proliferation and osteogenic differentiation while concurrently inhibiting chondrogenic and adipogenic differentiation (Brandt et al., 2018). Moreover, the combination of IFN-γ and TNF-α can further significantly enhance their proliferation (Mohammadpour et al., 2016). In contrast, placenta-derived MSCs (PMSCs) demonstrate a concentration-dependent response: low concentrations of IL-1, IL-6, and IL-8 enhance proliferation, whereas high concentrations of IL-1β upregulate PD-L1 via the JAK/NF-κB pathway, thereby inhibiting their adhesion and proliferation (Li et al., 2007; Zhang A. et al., 2020). Comparative analyses further highlight these source-dependent disparities, revealing that the chondrogenic potential of both BMSCs and AD-MSCs in the presence of TGF-β3 exceeds that of annulus fibrosus-derived MSCs (AF-MSCs) (Park et al., 2011b). Furthermore, under inflammatory conditions, AD-MSCs display more pronounced proliferation enhancement and simultaneous chondrogenic inhibition compared to amniotic fluid-derived MSCs (AMSCs) (Borem et al., 2019), further underscoring the critical influence of cellular origin on MSC fate in a degenerative niche.

Besides cytokines, matrix metalloproteinases (MMPs) also play a key role in the inflammatory microenvironment. MMPs (such as MMP-1, -2, -3, -7, -9, -13, −14, and ADAMTS-4) are upregulated in IDD and correlate with the severity of degeneration (Huang YC. et al., 2013; Zhang C. et al., 2020), influencing MSC function by regulating ECM metabolism. Membrane-type MMP-1 (MT1-MMP) promotes BMSC proliferation, migration, and differentiation towards an NP-like phenotype via Wnt signaling (Neth et al., 2006; Ries et al., 2007; Lu et al., 2010). MMP-2 and MMP-9 also promote the proliferation and migration of UC-MSCs and AD-MSCs, respectively (Gao F. et al., 2016; He et al., 2019; Marquez-Curtis et al., 2014). Conversely, MMP-3 overexpression reduces collagen I levels in AD-MSCs (Rong et al., 2020), and its downregulation promotes NP-MSC chondrogenic differentiation (Zhang Q. et al., 2021). Studies also suggest that during MSC chondrogenic differentiation, MMP-3 expression decreases (An et al., 2010), and MMP-2 might be a potential marker for the differentiation process (Arai et al., 2016).

Unlike MSCs, which generally possess anti-inflammatory properties (Chen XX. et al., 2019; Pedrazza et al., 2017; Li and Zhao, 2016), disc progenitor cells are more susceptible to damage in inflammatory environments. IL-1β can induce apoptosis in rat NP progenitor cells, inhibit proliferation, and reduce Sox9 and aggrecan expression (Lyu, 2022); furthermore, inflammatory conditions may promote the differentiation of NP progenitor cells towards a neurogenic lineage, participating in the generation of discogenic pain (Navone et al., 2012).

In summary, the inflammatory microenvironment in IDD is extremely complex, with different cytokines and proteases regulating the survival, proliferation, migration, and differentiation fate of MSCs and disc progenitor cells in a concentration- and cell-dependent manner. A deep understanding of these effects and their mechanisms is crucial for optimizing MSC-based regenerative therapy strategies for the intervertebral disc.

## 7 Clinical trials of cell therapy for intervertebral disc regeneration

Although a clear biological foundation has not yet been established, direct tissue replacement therapies are gradually losing appeal. The biochemical interactions between transplanted cells and recipient cells are crucial, and elucidating the signaling pathways involved is both a challenging and critical task. Progress in evaluating cell therapies depends on an in-depth understanding of the disease itself and the treatment process. As clinical trials of cell therapies for intervertebral disc degeneration gain increasing attention, a comprehensive assessment of the biological elements of degeneration and regeneration has become particularly essential.

As of January 2025, 18 registered strategies involving MSC-based treatments for lumbar intervertebral disc degeneration have been documented (Table 4), providing important references for the development of cell therapies for disc degeneration. Among these, 13 clinical trials focus on bone marrow-derived MSCs (BM-MSCs), three studies investigate adipose-derived stem cells (ADSCs), and two other studies explore the use of umbilical cord-derived MSCs in the treatment of lumbar intervertebral disc degeneration.

**TABLE 4:** Clinical trials were investigating cell-based therapies for IVD degeneration.

Clinical trial identifier	Clinical phase	Country	Indication	Enrollment (original/Actual)	Source of MSCs	Follow-up	Year (study start)	Status
NCT03461458	Phase I, prospective, non-randomized, dose-escalation study	United States	Lumbar disc degeneration and chronic low back pain	16/1	Autologous adipose-derived mesenchymal stromal cells	24 months	2018	Terminated
NCT03692221	Early phase I, randomized, open-label study	United States	Symptomatic degenerated intervertebral disc disease	24/0	Autologous bone marrow derived mesenchymal stem cells	12 Months	2018	Not yet recruiting
NCT04735185	Interventional/Not applicable	United States	Discogenic back pain	106/106	Autologous bone marrow-derived mesenchymal stem cells (BMC)	12 Months	2021	Suspended
NCT02440074	Interventional/phase I-II	Location not provided	Lumbar degenerative disc disease	10/0	Autologous bone marrow derived mesenchymal stem cells	6 Months	2011	Withdrawn
NCT02338271	Interventional/Phase 1	South Korea	Lumbar disc degeneration and chronic low back pain	10/10	Autologous adipose derived stem cell	12 Months	2015	Unknown status
NCT04499105	Interventional/phase 2	Indonesia	Degenerative disc disease with no improvement from conventional treatment	10/10	Allogenic mesenchymal stem cells from the umbilical cord	6 Months	2017	Recruiting
NCT06589271	Interventional/Phase 1	China	Lumbar intervertebral disc degeneration	20/20	hUC-MSCs	12 Months	2024	Suspended
NCT04759105	Interventional/phase 2	Italy	chronic low back pain (LBP)	52/48	Autologous BM-MSC	12 Months	2019	Completed
NCT01860417	Interventional/Phase 1,2	Spain	Lumbar disc degeneration and chronic low back pain	24/24	Allogenic mesenchymal stem cells (MSV)	12 Months	2013	Completed,no results posted
NCT05066334	Interventional/phase 2	Italy	Low back pain	52/52	Autologous BM-MSC	12 Months	2021	Recruiting
NCT03737461	Interventional/phase 2,3	France	Lumbar disc degeneration and chronic low back pain	112/113	Allogeneic adult BM-MSC	12 Months	2019	Active, not recruiting
NCT04559295	Interventional/phase 2,3	United States	Disc degeneration and low back pain	80/80	Bone marrow concentrate stem cells (BMC)	24 months	2018	Active, not recruiting
NCT04414592	Open-label, single-arm study	China	Lumbar disc degeneration and chronic low back pain	20/20	hUCMSC	12 Months	2020	Recruiting
NCT01513694	Interventional/phase 1, 2	Spain	Lumbar intervertebral degenerative disc disease	15/15	Autologous mesenchymal stem cells	6 Months	2010	Completed
NCT01290367	Phase II, prospective, randomized, double-blind, controlled, multicentre study	United States	Lumbar disc degeneration and chronic low back pain	80/100	Adult mesenchymal precursor cells (MPCs)	36 Months	2011	Completed. no results posted
NCT01643681	Open-label, single-arm study	South Korea	Lumbar intervertebral disc degeneration	8/0	Autologous adipose derived mesenchymal stem cells	6 months	2012	Withdrawn
NCT03912454	Single-arm, prospective case series	United States	Lumbar disc degeneration and chronic low back pain	20/20	Autologous bone marrow aspirate concentrate (BMAC) injection	12 Months	2019	Enrolling by invitation
NCT03340818	Randomized, doubleblind, placebo-controlled study	United States	Chronic low back pain with abnormal Disc pathology	60/66	Bone marrow concentrate (autologous)	12 months	2018	Recruiting

## 8 Strategies to improve the therapeutic effect of MSCs for IVDD and their challenges in clinical application

The microenvironment plays a decisive role in MSC-mediated intervertebral disc regeneration. Whether for endogenous repair or exogenous transplantation, its efficacy is strictly regulated by the microenvironment. To improve treatment efficiency, current strategies mainly focus on three directions: first, enhancing the tolerance of MSCs to the degenerative microenvironment to maintain their regenerative capacity; second, preconditioning or remodeling the degenerative microenvironment to create favorable conditions for their function; third, developing cell-free therapeutic strategies based on the MSC secretome or extracellular vesicles. Recent studies have validated the feasibility of these directions, providing new pathways for advancing disc regeneration therapy.

### 8.1 Gene modification of MSCs

Genetic modification of mesenchymal stem cells (MSCs) using viral or non-viral vectors can induce the overexpression of functional proteins and soluble factors, thereby enhancing stemness, differentiation, migration and homing capabilities, immunomodulation, and other repair-related functions (*in vitro* and *in vivo*), and improving their resistance to harsh microenvironments and apoptosis. Researchers utilize a range of genes related to the therapeutic capacity of MSCs to improve cell survival and therapeutic efficacy. The SDF-1/CXCR4 chemotactic signaling axis plays an important role in regulating stem cell-based tissue regeneration. Wei et al. (2016) prepared MSCs overexpressing CXCR4 (CXCR4-MSCs) via lentiviral transfection, enhancing MSC migration and improving the efficiency of intervertebral disc (IVD) regeneration. Furthermore, Feng et al. (2020) designed a novel delivery system using nanofiber sponge microspheres loaded with rabbit-derived MSCs and anti-miR-199a, which continuously released anti-miR-199a to promote stem cell-mediated nucleus pulposus regeneration and inhibit calcification.

Additionally, SOX9 and TGFβ1, as key regulators maintaining disc homeostasis and promoting chondrogenesis (Lv et al., 2016; Liu C. et al., 2021), are receiving widespread attention in genetic engineering. Studies show that human umbilical cord-derived MSCs (hUC-MSCs) transfected with SOX9 and TGFβ1 have significantly enhanced chondrogenic capacity and effectively promote disc regeneration (Khalid et al., 2023). Building on this, Lee et al. (2025) used the CRISPR/Cas9 system to stably integrate SOX9 and TGFβ1 into the AAVS1 “safe harbor” locus of human MSCs, successfully engineering tonsil-derived MSCs (ToMSCs). These cells effectively enhanced ECM repair capacity and suppressed inflammation in degenerated discs. Similar studies also confirmed (Khalid et al., 2022) that hUC-MSCs transfected with Sox-9 and Six-1 significantly upregulated key genes such as TGF-β1, BMP, and aggrecan, exhibited superior homing and integration capabilities after transplantation, and successfully differentiated into functional nucleus pulposus cells. Furthermore, overexpression of the Bcl-2 and Wnt-11 genes has also been shown to significantly improve the survival and differentiation efficiency of stem cells in the harsh disc microenvironment (Fang et al., 2013; Chen et al., 2017).

Besides gene overexpression, regulation of stem cells can also be achieved through silencing approaches. Studies have shown that silencing the anti-chondrogenic factor miR-221 can efficiently promote human BMSC chondrogenesis *in vitro* and *in vivo*, even without the need for the chondrogenic inducer TGF-β (Lolli et al., 2016).

Although some gene therapy applications have shown initial success, and the emergence of new gene editing technologies has brought IDD treatment to a new starting point, many obstacles still need to be overcome, such as safety concerns, high cost, and low transfection efficiency.

### 8.2 Endogenous repair

Given the limitations of exogenous MSC/disc stem cell transplantation, strategies to activate endogenous nucleus pulposus-derived stem cells (NPMSCs) to participate in intervertebral disc degeneration (IDD) repair have become a research hotspot. This strategy aims to reduce NPMSC apoptosis and senescence, enhance their proliferation and differentiation capacity, thereby promoting endogenous repair.

Several studies focus on drugs and small molecule compounds that can enhance NPMSC tolerance to the degenerative microenvironment. Liang et al. (2018) found that cyclosporine A, an inhibitor of the mitochondrial permeability transition pore, could inhibit stress-induced NPMSC apoptosis by alleviating mitochondrial dysfunction and oxidative stress. Hu et al. (2019) reported that pioglitazone could also reduce stress-induced cell apoptosis by inhibiting oxidative stress and mitochondrial damage, a mechanism potentially related to the reduction of ROS and malondialdehyde production and the decrease in mitochondrial membrane potential. Huang et al. (2020) confirmed *in vitro* and *in vivo* that puerarin could alleviate stress-induced NPMSC apoptosis by activating the PI3K-Akt signaling pathway.

Furthermore, naringin could protect rat NPMSCs from hydrogen peroxide-induced apoptosis through ROS-mediated PI3K-Akt pathway activation (Nan et al., 2020a); 6-gingerol not only reduced the apoptosis rate and intracellular ROS levels in human NPMSCs but also protected the extracellular matrix, possibly related to autophagy and PI3K-Akt pathway activation (Nan et al., 2020b). Simvastatin, at appropriate concentrations, could promote NPMSC proliferation and induce the expression of NP cell-like ECM components, potentially via the hypoxia-inducible factor-1α signaling pathway (Huang et al., 2019). These studies suggest that some traditional Chinese medicine components and small molecule compounds hold promise for reducing NPMSC apoptosis under mechanical and oxidative stress.

Bioactive cytokines and enzymes have also been found to regulate NPMSC function. Tao et al. (2015) reported that transforming growth factor-β3 and insulin-like growth factor-1 could synergistically enhance the viability, differentiation capacity towards nucleus pulposus cells, and ECM synthesis in rat NPMSCs. Cheng et al. (2019) showed that low concentrations of tumor necrosis factor-α, while promoting rat NPMSC proliferation and migration, inhibited their differentiation towards nucleus pulposus cells, a process potentially involving the NF-κB signaling pathway. Another study found that heme oxygenase-1 could inhibit oxidative stress-induced NPMSC apoptosis and decreased viability, reduce ROS generation, through autophagy-related pathways (Chen et al., 2020). These results indicate that specific cytokines and enzymes play important roles in regulating the biological behavior of NPMSCs.

### 8.3 Microenvironment-based strategies

Addressing the issue of oxidative stress in IVDD, some studies have attempted to use various metal or non-metal nanomaterials to simulate endogenous ROS-degrading enzymes for ROS clearance. For example, Conley et al. (2023) and Yang et al. (2024) found that MnO2 nanosheets or nanoparticles could be used as ROS-scavenging materials. Similarly, Sun et al. (2024) reported that carbonized manganese-containing nanodots (MCD) made from manganese gluconate and L-aspartic acid could serve as nanozyme particles for ROS clearance. Lee et al. (2022) found that a manganese porphyrin analog, BuOE (MnTnBuOE-2-PyP5+), could be added as a superoxide dismutase mimetic to methacrylated chondroitin sulfate A microparticles to provide ROS-scavenging properties. Han et al. (2023) utilized cerium-modified mesoporous silica nanoparticles within a developed composite hydrogel system to achieve antioxidant properties. Furthermore, Zhou et al. (2024) designed and fabricated antioxidant polyphenol nanoparticles by leveraging the reactive oxygen species (ROS)-scavenging properties of polyphenol-based structures. Bai et al. (2020) developed ROS-unstable linkers that also possessed ROS-scavenging ability using a quaternization reaction of phenylboronic acid). As IVDD worsens, disordered glycolysis metabolism in disc cells increases lactic acid, making the microenvironment weakly acidic (pH 6.2–6.8). Inspired by this, some material studies attempt to reverse the environment or alter cellular responses to resist the acidic environment. For instance, Peng et al. (2024) immersed MnO2 nanozymes loaded with lactate oxidase into glucose-rich decellularized nucleus pulposus hydrogel microspheres, endowing them with the ability to improve cellular glucose metabolism and the acidic microenvironment while providing antioxidant effects. Based on biomimetic mineralization and microfluidic technology, Zheng et al. (2024) developed hydrogen ion-capturing hydrogel microspheres composed of mineralized TGFβ and catalase nanoparticles, neutralizing the acidic microenvironment by capturing excess hydrogen ions via the calcium carbonate mineralization layer. Considering the exacerbation of acidic stress on disc cells, Han et al. (2022) designed and synthesized acid-sensitive ion channel inhibitor Sa12b-delivering functionalized peptides that self-assemble under acidic conditions, enhancing the bioactivity of nucleus pulposus stem cells by inhibiting acid-sensing ion channels. Similarly, Bian et al. (2021) covalently conjugated the ASIC-3 acid channel inhibitor APETx2 to injectable hydrogel microspheres, combined with nucleus pulposus cells to create “peptide-cell-hydrogel” regenerative microspheres that inhibit acidic stimulation and local inflammatory cytokine storms, enabling controlled release for over 28 days in an acidic environment. Additionally, Xia et al. (2023) utilized esterase activation under acidic conditions to design esterase-responsive nanomicelles delivering ibuprofen, allowing on-demand regulation of the adverse effects of the local acidic environment on intradiscal cells.

Furthermore, the disc microenvironment contains abundant pro-inflammatory and pro-neurogenic factors. Inspired by this, Peng et al. (2023) loaded the hydrophobic core of amphiphilic polycarbonate cationic nanoparticles with the nerve growth factor receptor antagonist TrkA-IN-1 and incorporated the drug-loaded nanoparticles into a decellularized annulus fibrosus matrix (DAF) hydrogel, which could inhibit the formation of a chronic inflammatory microenvironment by pro-inflammatory factors and nerve growth factor-induced nerve ingrowth into the annulus fibrosus. Recently, Yang et al. (2024) using membrane coating technology, coated MnO2 nanoparticles with nerve growth factor receptor-overexpressing macrophage membranes, endowing them with the ability to bind inflammatory factors and nerve growth factor, effectively improving IVDD-related inflammation and pain.

### 8.4 Hydrogels

Hydrogels are three-dimensional polymeric materials with extensive hydrophilic structures, capable of providing a suitable aqueous environment for disc cells and promoting cell proliferation. Additionally, hydrogels possess adjustable biochemical and mechanical properties, giving them great prospects in the field of IDD treatment. Currently, using hydrogels as scaffolds to load seed cells is a widely applied therapeutic strategy. In animal studies of intervertebral disc degeneration, cell-based therapeutic strategies have shown that injecting mesenchymal stem cells (MSCs) into the nucleus pulposus can allow them to survive for months and induce ECM production. By loading seed cells, the number of nucleus pulposus cells within the disc can be directly supplemented, or they can be differentiated into NP-like cells, increasing the ECM content within the disc and delaying disc degeneration. This can also, to some extent, restore the mechanical properties of the disc. While providing mechanical strength, using hydrogels as scaffolds can also load cells and provide a good growth environment. This suggests that *in situ* injection of hydrogel-loaded bone marrow mesenchymal stem cells may be a clinically feasible, minimally invasive treatment strategy for intervertebral disc degeneration (Clark et al., 2020; Ukeba et al., 2020; Zeng et al., 2015; Chen P. et al., 2019; Luo et al., 2022; Yu et al., 2021).

Furthermore, in the field of intervertebral disc tissue engineering, cell-free therapeutic strategies based on hydrogels primarily include drug delivery, controlled release of cytokines, and gene therapy. The degenerative process of intervertebral discs is often accompanied by the overexpression of matrix metalloproteinases (MMP3, MMP13) and inflammatory factors (IL-1, IL-6, IL-8, PEG2, TNF-α) (Wang et al., 2020). To address this pathological characteristic, hydrogels have emerged as ideal intervention carriers due to their superior controlled-release capabilities. For example, aspirin-loaded hydrogels can alleviate postoperative inflammation (Liu Y. et al., 2021). Rapamycin-loaded hydrogels reduce ROS levels and promote M2 macrophage polarization (Bai et al., 2020), while bevacizumab-incorporated thermosensitive hydrogels delay IDD progression by inhibiting VEGF expression (Chen Q. et al., 2022). In the realm of growth factor delivery, thiol-modified hyaluronic acid hydrogels enable sustained release of PDGF-BB, improving degenerative markers (Masuda and An, 2004). Additionally, pullulan microsphere-cellulose composite hydrogels facilitate the prolonged release of TGF-β1 and GDF-5 (Henry et al., 2017). Regarding gene therapy, aldehyde hyaluronic acid/siRNA composite hydrogels enable targeted intervention in the STING signaling pathway (Chen J. et al., 2022). Thermosensitive poloxamer hydrogels can also control the release of rAAV-sox9 vectors to enhance chondrogenic capacity (Madry et al., 2020). Collectively, these studies demonstrate the significant potential of hydrogels as multifunctional delivery systems in the treatment of intervertebral disc degeneration.

Single components often fail to meet the complex structural and functional needs of the intervertebral disc, while multiple components assembled through certain strategies often achieve higher therapeutic effects than single components. Therefore, researchers are committed to combining multiple bioactive substances to achieve synergistic therapeutic effects. Kim et al. (2020) paired human bone marrow mesenchymal stem cells with TGF-β3, creating a favorable environment for chondrogenic differentiation, and effectively induced disc regeneration in a canine model; Ma et al. (2024) combined growth factors, NPMSCs, and hydrogels, demonstrating good repair effects on rat IDD *in vitro* and *in vivo*; Mohd Isa et al. (2023) developed a three-dimensional hydrogel based on NPC extracellular matrix components, successfully guiding human mesenchymal stem cells to differentiate into NPCs. Sun et al. (2021) used polycaprolactone material as a mechanical support framework, loaded with CTGF or TGF-β3 polydopamine nanoparticles and mixed with mesenchymal stem cells (BMSCs) to promote nucleus pulposus and annulus fibrosus cell regeneration. Xia et al. (2023) used esterase-responsive copolymer ibuprofen-PEG-PIB nanomicelles to modify progenitor cells, thereby achieving a synergistic effect of ibuprofen on the transplanted progenitor cells. These studies all indicate that combined therapeutic strategies integrating multiple active components through hydrogels show significant advantages over single therapies, making hydrogels a promising biomaterial in IDD treatment.

### 8.5 Cell-free strategies

In recent years, researchers have discovered that extracellular vesicles (EVs) secreted by stem cells, such as exosomes and microvesicles, possess immunomodulatory functions similar to stem cells (Ha et al., 2020). EVs are membrane-bound particles released by cells and belong to one component of the stem cell secretome (Elahi et al., 2020). According to physical size, nanoscale EVs are defined as small extracellular vesicles. They can regulate the phenotype of target cells and are important mediators of intercellular communication (Mendt et al., 2019; Yu et al., 2014). Currently, stem cell vesicle therapy is gradually developing as an alternative to cell therapy and has gained widespread attention in intervertebral disc degeneration (IDD) repair.

As a “cell-free” therapeutic system, EVs exhibit good stability both *in vitro* and *in vivo* and possess strong tolerance to adverse microenvironments (Wu P. et al., 2018). Their carried active components, such as proteins, nucleic acids, and lipids, are protected by a bilayer membrane structure and can be delivered to target cells via membrane fusion, participating in intercellular communication (Chang et al., 2018; Keshtkar et al., 2018). Compared to mesenchymal stem cells (MSCs), EVs are easier to isolate from cell supernatants, have higher yields, lower costs for preparation, storage, and transportation, and carry no risk of malignant differentiation (Zhao et al., 2019). Overall, MSC-derived EVs have multifaceted application advantages; however, their functional mechanisms are not fully elucidated, and clinical application strategies still require systematic optimization.

As key mediators of intercellular communication, EVs can deliver bioactive molecules such as nucleic acids, proteins, and lipids to recipient cells, thereby regulating cell metabolism, microenvironment homeostasis, and physiological functions (Gao et al., 2018). The cargo of EVs can reflect the pathological state of the source cells, covering different disease types and stages (Kalluri and LeBleu, 2020), thus holding potential value in the diagnosis and treatment of various diseases, including IDD. Studies show that MSC-EVs can alleviate local inflammation by inhibiting inflammatory responses and reducing the release of inflammatory factors (Harting et al., 2018). Simultaneously, MSCs-EVs promote the proliferation and differentiation of disc cells, enhance the synthesis of collagen and other ECM components, and drive tissue reconstruction (Lan et al., 2019). Moreover, MSC-EVs play a crucial role in mediating communication between MSCs and nucleus pulposus (NP) cells. They have been shown to not only promote NP cell proliferation and enhance metabolic function (DiStefano et al., 2022), but also improve cell survival through the regulation of gene expression (Zhu L. et al., 2020). Furthermore, by delivering antioxidant proteins, MSC-EVs can inhibit NP cell apoptosis, thereby delaying the progression of intervertebral disc degeneration (IDD) in organ culture models (Liao et al., 2019). The miRNAs carried within MSC-EVs are closely associated with the initiation and progression of IDD, suggesting their potential as novel therapeutic targets (Zhu G. et al., 2020).

Although the therapeutic advantages of EVs are evident, there are still shortcomings in practical application, such as low therapeutic efficiency and rapid degradation rates (He et al., 2018). Natural EVs contain limited amounts of bioactive substances, and due to the complex pathogenesis of diseases, the targeting of EV therapy still needs improvement (Sterzenbach et al., 2017; Wang X. et al., 2018). Furthermore, during cellular uptake and metabolic absorption, EVs themselves degrade quickly or are actively cleared by the body, leading to a high degradation rate (Luan et al., 2017). In response to these issues, EVs are increasingly being functionalized using engineering strategies, including membrane modification and cargo loading. This functionalization enables engineered EVs to achieve targeted therapy or exhibit enhanced stability (Bellavia et al., 2018; Tran et al., 2020).

To further enhance therapeutic efficacy, engineered extracellular vesicle technology has emerged. This technology involves genetic modification of parent cells, membrane modification, or *in vitro* modification of isolated vesicles to enhance their therapeutic efficacy or compensate for the limitations of natural EVs. Engineering strategies mainly include two directions: *in vivo* modification and *in vitro* modification.

#### 8.5.1 *In vivo* modification

Genetic engineering of parent cells is currently the most commonly used method for *in vivo* editing. For example, introducing exogenous mRNA into parent cells can be packaged into EVs and then delivered to target cells to induce specific gene expression (Zomer et al., 2015; Ridder et al., 2014). Similar strategies also include loading non-coding RNAs such as miRNA or small interfering RNA into EVs (Akao et al., 2011; Yang et al., 2011). Furthermore, through fusion gene design, target proteins (such as targeting peptides) can be enriched on the EV membrane surface, effectively enhancing their tissue targeting. Researchers have successfully achieved neural targeting of EVs using the fusion expression of the RVG peptide and the vesicle membrane protein LAMP2B (Tian et al., 2014; Alvarez-Erviti et al., 2011). Some scholars have also used the more stable glycosylphosphatidylinositol (GPI) anchor system instead of LAMP2B to improve expression stability (Kooijmans et al., 2016). Other fusion sites such as the PDGF receptor (Ohno et al., 2013) and the lactadherin C1C2 domain (Rountree et al., 2011) have also been widely used in the construction of functionalized EVs.

#### 8.5.2 *In vitro* modification

*In vitro* modification is performed directly on purified EVs, offering advantages such as operational controllability and high yield. Membrane modification strategies include covalent conjugation methods, such as bioconjugation and click chemistry reactions, as well as non-covalent approaches like multivalent electrostatic interactions or receptor-ligand binding. For instance, the association of cationic lipids with EV membranes can enhance cellular uptake efficiency (Nakase and Futaki, 2015), while transferrin-conjugated superparamagnetic particles can endow EVs with magnetic responsiveness for imaging-guided therapy (Correia Carreira et al., 2016; Neubert and Glumm, 2016; Silva et al., 2015). In terms of cargo loading, membrane permeabilization techniques including electroporation, saponin treatment, and osmotic dialysis are widely employed to load exogenous siRNA, small-molecule drugs, and other therapeutic agents into EVs (Fuhrmann et al., 2015; Wassler et al., 1987; Gutierrez Millan et al., 2004). Although these methods may cause some degree of membrane disruption, optimization of experimental conditions can effectively preserve EV integrity and functionality.

In summary, engineered extracellular vesicles, through precise functional design, show significant advantages in improving targeting, optimizing drug loading, and enhancing therapeutic effects, providing new technological pathways for the treatment of intervertebral disc degeneration. With in-depth research on their biological characteristics and engineering methods, EVs are expected to become the next-generation therapeutic strategy replacing stem cell therapy.

## 9 Standards need to be developed for clinical applications

Over the past few decades, research and therapy based on mesenchymal stem cells (MSCs) have made significant progress due to their advantages such as immune evasion capability, diversity of source tissues, ease of isolation, rapid expansion, and tissue repair and immunomodulatory functions. However, the development of MSC products has been relatively slow, partly due to the lack of precise standards. Numerous key parameters affect the efficacy of MSCs, covering aspects such as the type of transplanted cells, cell source, culture and expansion conditions, transplantation route, cell number, patient selection, and efficacy evaluation indicators.

### 9.1 Cell type

In intervertebral disc regeneration therapy, the choice of ideal cell type requires comprehensive consideration of its adaptability, function, and availability. Nucleus pulposus cells, while theoretically most advantageous due to their ability to adapt to the harsh intradiscal environment and synthesize specific matrix, are limited in number in degenerated discs, their acquisition is invasive, and they are difficult to use for repairing the annulus fibrosus and endplate, limiting clinical application. Allogeneic disc cells or functionally similar chondrocytes can serve as alternative options. MSCs have become a research hotspot due to their multidifferentiation potential and immunomodulatory capabilities (Naji et al., 2019). Although small-scale clinical trials have confirmed their safety, efficacy remains limited. The future requires further screening for the optimal cell source, while considering accessibility, environmental adaptability, and duration of action.

### 9.2 Cell leakage problem

The intervertebral disc is an avascular tissue with low cell clearance; however, substantial cell loss occurs following transplantation (Vadala et al., 2012). The primary causes of leakage include reduced cell adhesion due to high osmotic pressure and physical damage to the annulus fibrosus. Injection volume must also be carefully controlled, as animal studies indicate that reducing the volume can lower the risk of nucleus pulposus protrusion. Additionally, alternative delivery routes such as transvertebral or adjacent vertebral body approaches remain under investigation.

### 9.3 Cell source

The selection of cell sources necessitates a careful trade-off between the advantages and disadvantages of autologous versus xeno-/allogeneic cells. Autologous cells present a low risk of immune rejection but involve complex harvesting procedures, whereas allogeneic cells also carry a relatively low immunological risk owing to the immune-privileged nature of intervertebral discs, in addition to offering the advantage of scalable production (Ganguly et al., 2017). In clinical trials, neither allogeneic chondrocytes nor MSCs have triggered significant immune responses; however, their therapeutic efficacy remains subject to individual variability.

### 9.4 Number of transplanted cells

In existing studies, the number of cells injected per disc ranges from 1 × 10^6^ to 132 × 10^6^, and the optimal dose has not reached a consensus. Some studies suggest a positive correlation between dose and efficacy, but subsequent studies have found no difference (Centeno et al., 2017; Kumar et al., 2017). Excessive transplantation may lead to nutrient competition exacerbating degeneration, while insufficient numbers may fail to achieve effective repair. Therefore, establishing a reasonable range for the number of transplanted cells, considering the degree of disc degeneration, is necessary to promote clinical translation.

### 9.5 Patient selection

The success of cell therapy largely depends on the precise selection of patients. It is necessary to define the applicable population and the intervenable stage of disc degeneration, rather than surgical indications. The ideal treatment timing is the early stage of degeneration, when the disc structure is relatively intact (annulus fibrosus not ruptured, endplate not calcified), and functional cells remain. Accurately identifying this stage relies on more advanced examination techniques to non-invasively and rapidly assess the intradiscal structure and biological status. To control research heterogeneity, patient inclusion should be limited to specific, well-defined indications. Future clinical research will still be needed to establish non-invasive assessment systems for accurate judgment of pain sources, immune microenvironment, and regenerative potential.

### 9.6 Efficacy evaluation standards

The heterogeneity in the efficacy of cell transplantation is largely due to the lack of unified evaluation standards. Existing evaluations mostly rely on subjective clinical symptom scores or semi-quantitative imaging indicators using MRI and X-rays, making it difficult to accurately reflect the actual function of transplanted cells. Although quantitative MRI techniques can provide more objective assessment data, their results still lack histological validation (Smith et al., 2018; Thorpe et al., 2018). Therefore, establishing an objective, unified, and biologically meaningful efficacy evaluation system is key to accurately assessing the value of cell therapy.

## 10 Conclusion and future perspectives

MSCs have emerged as the most promising cellular therapeutic candidates for IVDD due to their distinctive advantages. However, various physicochemical and biological factors within the IVD microenvironment, such as pH, osmotic pressure, hypoxia, inflammation, and mechanical stress, severely compromise the survival and function of transplanted cells. Consequently, the systematic regulation of this microenvironment is a critical prerequisite for clinical translation. Existing clinical trials have demonstrated encouraging outcomes, leading to the development of various efficacy-enhancing strategies, including tissue engineering, genetic modification, endogenous repair activation, and cell-free therapies. Nevertheless, the efficacy and safety of these strategies require further in-depth validation, and their clinical translation potential remains to be clarified. Concurrently, multiple aspects ranging from cell preparation to clinical intervention, encompassing cell quality, type selection, dosage regimens, and personalized efficacy assessment, necessitate systematic optimization.

To overcome these bottlenecks and advance this therapy towards clinical application, stem cell-based regenerative treatment for IVD is entering a new phase, with future efforts focusing on the following key directions:

First, personalized therapy will become the cornerstone. Given the high heterogeneity of IVDD, it is essential to utilize novel biomarkers or radiomics for precise subtyping based on a patient’s specific microenvironmental characteristics, such as inflammation level or acidosis severity. This will enable the matching of the most suitable stem cell type, engineered vesicles, or composite biomaterials for each individual. Second, multimodal combination therapy represents the inevitable path to breakthrough efficacy. Integrating multiple intervention modalities to simultaneously address the multifaceted pathological factors within the degenerative microenvironment is crucial. For instance, combining genetically-enhanced MSCs with smart materials capable of modulating the microenvironment, or synergizing engineered extracellular vesicles with pathology-responsive scaffold systems, can achieve superimposed and optimized therapeutic effects. Third, technological innovation is the fundamental driver of field progression. This includes the development of next-generation biomaterials capable of dynamically responding to the pathological microenvironment, such as enzymes, pH, or mechanical forces, and the establishment of standardized, scalable production platforms for engineered extracellular vesicles to ensure quality control and targeted delivery. Finally, successful clinical translation hinges on internationally unified quality standards and clinical protocols. This involves defining Critical Quality Attributes for therapeutic products, adopting clinical trial designs based on precise stratification, and implementing objective efficacy endpoints, thereby translating the potential of basic research into definitive clinical treatments.

In summary, by focusing on the four major directions of personalization, combination strategies, technological innovation, and translational research, this field holds the promise to fundamentally reshape the therapeutic landscape for intervertebral disc degeneration.
